# Apprising Diagnostic and Prognostic Biomarkers in Cutaneous Melanoma—Persistent Updating

**DOI:** 10.3390/jpm12091506

**Published:** 2022-09-14

**Authors:** Simona Roxana Georgescu, Cristina Iulia Mitran, Madalina Irina Mitran, Clara Matei, Carolina Constantin, Monica Neagu, Mircea Tampa

**Affiliations:** 1 Department of Dermatology, “Carol Davila” University of Medicine and Pharmacy, 050474 Bucharest, Romania; 2 Department of Dermatology, “Victor Babes” Clinical Hospital for Infectious Diseases, 030303 Bucharest, Romania; 3 Department of Microbiology, “Carol Davila” University of Medicine and Pharmacy, 050474 Bucharest, Romania; 4 “Cantacuzino” National Medico-Military Institute for Research and Development, 011233 Bucharest, Romania; 5 Immunology Department, “Victor Babes” National Institute of Pathology, 050096 Bucharest, Romania; 6 Colentina Clinical Hospital, 020125 Bucharest, Romania; 7 Faculty of Biology, University of Bucharest, 030018 Bucharest, Romania

**Keywords:** melanoma, biomarker, immune cells, microRNA, inflammation, metabolism

## Abstract

The incidence of melanoma, a very aggressive skin cancer, has increased over the past few decades. Although there are well-established clinical, dermoscopic and histopathological criteria, the diagnosis is often performed late, which has important implications on the patient’s clinical outcome. Unfortunately, melanoma is one of the most challenging tumors to diagnose because it is a heterogeneous neoplasm at the clinical, histopathological, and molecular level. The use of reliable biomarkers for the diagnosis and monitoring of disease progression is becoming a standard of care in modern medicine. In this review, we discuss the latest studies, which highlight findings from the genomics, epitranscriptomics, proteomics and metabolomics areas, pointing out different genes, molecules and cells as potential diagnostic and prognostic biomarkers in cutaneous melanoma.

## 1. Introduction

Melanoma is the most frequent of the aggressive cutaneous malignancies, being the first cause of death for patients with skin cancer [1]. Cutaneous melanoma develops from melanocytes and can be classified into four major types, superficial spreading melanoma (70%), the most prevalent form, nodular melanoma (15–30%), lentigo maligna melanoma (4–10%) and the less common form, acral lentiginous melanoma (<5%) [2]. However, it should be kept in mind that melanoma can develop wherever there are melanocytes appending to tissues; thus, it can develop in the skin, eye, nasopharynx or gastrointestinal tract [2]. The main risk factors associated with melanoma are phototypes I-II, an increased number of nevi, history of sun exposure, advanced age and family history of skin cancer [3,4,5]. Melanoma is considered the tumor with the highest mutation rate. Mutations in the *BRAF* gene are present in 50–60% of melanomas and the *CDKN2A* gene is altered in 16–41% of melanomas [6]. Other mutated genes associated with melanoma development are *NRAS* and *C-KIT* [7]. The intimate mechanisms of melanoma development are complex and still incompletely elucidated [8,9,10]. The main intracellular signaling pathways described as being involved in its pathogenesis are the MAPK, PI3K-AKT, MITF, NF-kB and Wnt pathways [11]. Although several markers have been described and algorithms for the rapid diagnosis of melanomas have been established, the tumors are often diagnosed in their late stages and the prognosis is poor in that phase of evolution [12]. In the present paper, we present the current research that focuses on identifying the best biomarkers for the diagnosis and prognosis of melanoma and the latest advances on this topic. The development of the cancer genomic datasets has led to improvements in the management of melanoma. These datasets are continuously updated and accelerate the understanding of the molecular basis of melanoma. In the first part of this review, we present the latest genetic profiles that can refine the diagnosis of melanoma and predict patient outcome. Identifying the effects of the alterations of the miRNA profiles brings new insights into the regulation of gene expression. Considering the dynamics of research in this field, new miRNAs have been proposed and we have summarized them in the present work. It is well known that the tumor microenvironment represents an important source of biomarkers in cancer. However, most research has focused on the classic markers, such as immune-associated cells in the tumor microenvironment, pro- and anti-inflammatory cytokines and growth factors. Analysis of markers such as lymphocyte cytosolic protein 2, autophagy and beclin 1 regulator 1 and loricrin opens new perspectives in understanding the role of the tumor microenvironment in melanoma progression. Furthermore, in addition to the conventional factors involved in angiogenesis, such as vascular endothelial growth factor, new factors, including angiopoietin-2 and the urokinase plasminogen activator receptor, have gained greater attention. Additionally, in this review, we highlight markers such as nicotinamide N-methyltransferase and TBC proteins that show promising potential as diagnostic markers in melanoma.

## 2. Materials and Methods

We have performed a non-systematic review by browsing the PubMed and Google Scholar databases. We have selected articles written in English published in the last five years (July 2017–July 2022), based on their relevance and our expertise in the field. Search terms included “melanoma” and “diagnostic biomarker” or “prognostic biomarker” or “biomarker”. We have excluded the articles that evaluated types of melanoma other than cutaneous melanoma and biomarkers other than those related to the diagnosis and prognosis of melanoma. We have focused on the main domains that develop biomarkers that are applicable in the management of melanoma, including genetics, epigenetics, tumoral tissue, systemic environment, inflammation and metabolism.

## 3. Novel Genetic Aspects in Melanoma

Melanoma exhibits significant heterogeneity, with diverse genomic alterations being one of the highest mutated tumors. Therefore, tumor genetic heterogeneity is an important player in the process of immune evasion, invasion and metastasis of new tissues. This genetic plasticity interferes with the proper diagnosis and treatment of melanoma [13]. Currently, melanoma staging is based on clinical and histopathological characteristics, but to identify new prognostic markers, it is important to investigate gene expression in the case of each melanoma patient [14], as new technologies have opened an entire new research area in genomic biomarkers in melanoma [15,16].

Data from The Cancer Genome Atlas (TCGA) and Gene Expression Omnibus (GEO) databases provide new genetic profiles aimed to predict patient outcomes. It was identified that downregulation of the genes *CDCA8* and *DPF* and upregulation of the genes *ABCC3*, *CAPS2*, *CCR6*, *CLU*, *PTK2B*, *SATB1*, and *SYNE* represent indicators for a positive prognosis of cutaneous metastatic melanoma [14]. Another study, using the same databases, showed that the over-expression of the following six hub genes, *CDC20*, *GNB2*, *PPP2R1A*, *AURKB*, *POLR2E*, and *AGTR1*, is associated with low overall survival [17]. Gao et al. proposed six genes, including *IQCE*, *RFX6*, *GPAA1*, *BAHCC1*, *CLEC2B*, and *AGAP2*, as candidate genes for melanoma prognosis [18]. Liu et al. showed that melanoma cells are characterized by low expression of fibulin 1 (FBLN1) and its low expression is associated with a poor outcome. By acknowledging that FBLN1 is involved in maintaining the junctions between cells and extracellular skeleton construction [19], its low expression can contribute to an accelerated metastatic process.

Gene expressions influence the tumor microenvironment that plays an important role in the progression and prognosis of melanoma. Yingjuan et al. analyzed genes related to stromal and immune cells in melanoma patients using TCGA database. They identified 10 genes (*IL15*, *CCL8*, *CLIC2*, *SAMD9L*, *TLR2*, *HLA.DQB1*, *IGHV1–18*, *RARRES3*, *GBP4*, *APOBEC3G*) involved in processes such as inflammation and immune response, which differentiate melanoma patients according to survival rate into two classes, with low and high risks [12]. Yan et al. identified four genes (*NOTCH3*, *DBN1*, *KDELC2*, and *STAB1*) that support the infiltration of pro-tumoral M2 macrophages in melanoma and even validated a prognostic risk score to evaluate mortality in patients with melanoma, based on the expression of these genes [20]. Li et al. identified *RTP4* (genes involved in the expression of interferon-responsive proteins) as a novel gene associated with cutaneous melanoma and revealed that there is a link between *RTP4* and tumor inflammatory infiltrate [21]. Another recent study analyzed 84 genes involved in cancer-related inflammation and immune response and highlighted a greater magnitude of the fold change in the CC chemokine ligand 18 (CCL18) gene (C-C motif) in plasma circulating cell free messenger ribonucleic acid from patients with melanoma with high disease burden, compared to those with low disease burden. The same results were obtained for the genes that encode the chemokine receptor (CCR) 1 and CCR4. Plasma transcriptomic levels of cluster of differentiation (CD) 274were also identified as increased in patients with progressive disease. The authors concluded that the plasma transcriptomic profile could represent a potential biomarker for melanoma [22].

The role of the hedgehog pathway in basal cell carcinoma is well known. In fact, the hedgehog (HH) pathway has been described as being involved in several malignant tumors, including melanoma [23,24]. Recently, Dunjic et al. analyzed whether there is a link between genetic polymorphisms in the sonic hedgehog (SHH) pathway and melanoma risk and identified a significant correlation between mutations in *GLI1 rs2228224 G* and *GLI1 rs2228226 G* alleles and susceptibility to melanoma. Moreover, the variant mutant genotype GG of *GLI1 rs2228226* polymorphism was associated with a lower survival rate [25]. In line with this, Peng et al. showed that blocking the HH pathway in melanoma cells was correlated with inhibition of cell growth and aggressiveness and upregulation of apoptosis. The study was performed on melanoma A375 cells using cyclopamine as an inhibitor of the HH signaling pathway [26]. Moreover, a crosstalk between the HH-GLI and ERK5 pathways has been described. GLI upregulates the expression of ERK5 in melanoma cells. GLI1 binds to the *MAPK7* promoter, the gene encoding the ERK5 protein. Inhibition of the HH-GLI and MEK5-ERK5 pathways can display an important antitumor effect [27]. Therefore, genes that can be markers for melanoma prognosis can also become future therapy targets.

## 4. miRNA Signature in Melanoma

In recent years, epitranscriptomics has gained its place in the biomarker domain, along with the important progress made in the field of sequencing methodologies [28]. Noncoding RNAs are among the most promising biomarkers in cutaneous melanoma in terms of tumor progression and recurrence rate; one of these, miRNAs, represents the class on which most studies have focused in the last decade [29]. miRNAs modulate the activity of both tumor and immune cells [30]. Ghafouri-Fard et al. elaborated an extensive list that includes numerous miRNAs that are upregulated or downregulated in melanoma [31]. miR-214, miR-148a, miR-221, miR-16, miR-29c, miR-146a-5p, miR-205, miR-203, miR-148, miR-155, miR-182, miR-200c, miR-211, miRNA-222 and miR-106b are the main miRNAs identified in the tumor samples or in the blood of patients with cutaneous melanoma [32]. Several miRNAs have been shown to distinguish healthy subjects from melanoma patients and melanoma patients without metastases from patients with metastatic melanoma. Thus, the role of miRNAs in improving the current approach for predicting the prognosis and guiding certain therapies was suggested [31]. However, Korfiati et al. conducted a review on the role of miRNAs in cutaneous melanoma and concluded that the results available so far are inconsistent, which may be the consequence of the various methodologies used and suggested that studies on a larger number of patients, along with improved bioinformatic tools, are required [29].

The investigation of miRNAs in melanoma is currently ongoing. In recent years, several new miRNAs related to the diagnosis and prognosis of melanoma have been reported. Downregulation or upregulation of certain miRNAs can predict patient outcomes. Sun et al. described miR-431 as a potential prognostic biomarker in melanoma. Low miR-431 expression in melanoma cells indicates tumor ulceration and is associated with a low survival rate [33]. Low expression of miR-107 was also correlated with a poor prognosis, with the lowest level being identified in metastatic melanoma [34]. miR-135b is a novel miRNA that was found to be upregulated in melanoma cells and it can promote cell proliferation and invasion [35]. Additionally, statistically significant correlations were identified between miR-424 expression and tumor characteristics (thickness, stage, ulceration, and metastasis). Increased expression of miR-424 is associated with decreased overall survival and disease-free survival [36].

Several studies have focused on identifying panels of miRNAs, not just one type of miRNA. Lu et al. identified a group of five miRNAs (miR-25, miR-204, miR-211, miR-510, miR-513c) that can be used to predict prognosis in melanoma patients. They identified a positive correlation between miR-204 and the prognosis of the disease, while the other four miRNAs correlated negatively with the prognosis [37]. These results are consistent with the research of Galasso et al., who pointed out the role of miR-204 in melanoma progression [38]. Another recent study reported four miRNAs (miR-142-5p, miR-550a, miR-1826, and miR-1201) that could be potential biomarkers for the development of primary melanoma [39]. Sánchez Sendra et al. analyzed circulating free miRNAs and identified that low circulating levels of miR-182-5p, and high circulating levels of miR-199a-5p, miR-877-3p, miR-1228-3p and miR-3613-5p indicate advanced melanoma and this panel may be used to detect micrometastatic regional lymph node disease [40].

It should be taken into account that miRNAs are degraded by RNA enzymes, but if they are packed in exosomes, degradation becomes more difficult. Thus, exosomes can be used in disease detection because they represent a simple method with good sensitivity and, moreover, are a stable family of molecules that can be identified in body fluids [41,42]. Tengda et al. identified significant differences between exo-miRNA-532-5p and exomiR-106b levels when they compared melanoma patients and healthy subjects. Moreover, they demonstrated that the two miRNAs have discriminatory power between patients with and without metastases [42]. Guo et al. also studied exosomal miRNA in melanomas and highlighted a negative correlation between the miR-1180-3p level and pro-metastatic processes, such as migration and cell invasion, concluding that the detection of exosomal miR-1180-3p in plasma from patients diagnosed with melanoma can be a promising tool for the diagnosis of melanoma [41]. The domain of miRNAs in melanoma biomarkers is still an intriguing and open-to-research subject and Table 1 summarizes the novel miRNAs or miRNA panels that can be good candidates for future development as biomarkers.

## 5. Tumor Microenvironment—A Source of Biomarkers

The tumor microenvironment (TME) comprises a plethora of cells and molecules, including malignant cells, tumor vessels, stromal cells, extracellular matrix (ECM), signaling molecules, etc., that are interconnected and actively contribute to tumor progression, resistance to therapy and immunosurveillance. The main non-cancer stromal cells are represented by endothelial cells, pericytes, immune cells, fibroblasts, cancer associated fibroblasts (CAFs), activated adipocytes, and mesenchymal stem cells (MSCs) [43]. In melanoma, the TME is characterized by significant plasticity, which facilitates the adaptation of cancer cells to various conditions [44]. Thorough analyses of the TME may provide new biomarkers that can be validated for melanoma prognosis and diagnosis.

Tumor-associated macrophages (TAMs) are a key component of the inflammatory infiltrate and have one of the most important roles in tumorigenesis. It is known that a high amount of TAMs in the TME is associated with a poor outcome. The identification of TAMs’ subtype is of outmost importance, as M1 macrophages exhibit anti-tumor effects, whereas M2 macrophages are involved in tumor growth [45]. M2 macrophages accumulate especially in the advanced stages of melanoma, but have also been identified in the early stages and their accumulation is correlated with tumor progression [46]. Other important cells involved in the maintenance of the TME are activated fibroblasts, namely cancer associated fibroblasts (CAFs); these cells exhibit a different phenotype from the fibroblasts encountered in healthy tissues. Tumor cells, by the paracrine effect, contribute to the differentiation of stromal fibroblasts into CAFs, which contain an increased amount of highly contractile protein (smooth muscle actin), similar to myofibroblasts. CAFs express a series of markers such as fibroblast-specific protein-1 (FSP-1), fibroblast-activating protein (FAP), platelet-derived growth factor receptor (PDGFRβ), tenascin-C, desmin and release numerous cytokines and chemokines, growth factors, and enzymes that contribute to melanoma invasion capacity [47].

Immune cells, key players of TME, have sub-populations that are involved in the pro-tumoral processes and can constitute important cellular biomarkers. Regulatory T cells (Tregs) induce an immunosuppressive microenvironment and contribute to tumor immune escape. Studies performed on murine models revealed that the transient depletion of Treg enhances the antitumor immunity and leads to a better clearance of the tumor [48]. In a small study, Sabbatino et al. demonstrated that the number of infiltrating CD8+ and FOXP3+ T cells, as well as the CD8+/FOXP3+ T cell ratio, can be used as prognostic biomarkers in thin melanoma. A low level of peritumoral or intratumoral CD8+ T cells and a peritumoral infiltrate rich in FOXP3+ T cells negatively correlate with disease-free survival [49]. On the contrary, tumor-infiltrating lymphocytes (TILs) have been shown to be crucial in inhibiting tumor progression. The melanomas that are characterized by a diffuse infiltrate have a better prognosis compared to the tumors in which the infiltrate is focal or absent. Furthermore, in advanced melanoma, it was noted that the inflammatory infiltrate is poorly represented [50].

Using the TGCA and GEO databases, Wu et al. have identified three main tumor-infiltrating immune cells, CD8+ T cells, M0 macrophages and M1 macrophages, which can modulate melanoma progression. The analysis has revealed that CD8+ T cells and M1 macrophages operate as protective factors, whereas M0 macrophages display a deleterious effect. In the same study, four immune-related genes, *HLA-B*, *HLA-DRA*, *CCL8*, and *IL2RA*, have been shown to be associated with a good prognosis in melanoma [51]. Wagner et al. showed that the immune cells in the TME express S100A8/A9, and the number of positive cells is higher in metastatic primary melanomas compared to non-metastatic melanomas. Furthermore, high serum levels of S100A8/A9 indicate the tumor metastasizing capacity. Thus, TME-associated S100A8/A9, a ligand of the receptor for advanced glycation end products (RAGE), which is upregulated in melanoma, may represent an important biomarker for prognosis [52]. However, changes in the epidermal TME may also play an important role in melanoma progression. Determining the expression of autophagy and beclin 1 regulator 1 (AMBRA1) and loricrin, two markers of epidermal differentiation, in the peritumoral epidermis can be useful in the further stratification of patients with AJCC stage I melanomas, as the decrease in AMBRA1 and loricrin expression is correlated with a high risk of metastasis [53].

Tagawa et al. suggested that the leucine zipper transcription factor ATF-like 2 (BATF2), a type I IFN-inducible transcription factor, can be a biomarker of invasiveness in melanoma. Altered tumor cells in the advanced stages of melanoma release Toll-like receptor ligands, such as damage-associated molecular patterns that induce BATF2 expression [54]. Lymphocyte cytosolic protein 2 (LCP2) is an actin binding protein encoded by the *LCP2* gene located on chromosome 5q33. Under physiological conditions, the protein is expressed by hematopoietic cells; however, malignant cells have been shown to express the protein, as in [55]. *LCP2* mediates, in particular, the development and activation of T cells through the TCR signaling pathway, but it also regulates the function of other cells, such as natural killer (NK) cells [56]. LCP2 belongs to the SLP-76 family of adapter proteins that are involved in numerous signaling pathways, and alterations in genes encoding the SLP-76 family of adapter proteins are associated with phenotypic changes, indicating their role in cell development and activity [57]. Wang et al. showed that *LCP2* influences the infiltration levels of immune cells in melanoma, especially CD8+ T cells, and the increased expression of *LCP2* is correlated with good overall survival in patients diagnosed with cutaneous metastatic melanoma. Moreover, considering that LCP2 is involved in the regulation of several immune pathways, it can be used as a prognostic marker for progression-free survival in patients treated with anti-PD-1 immunotherapy [56]. TME is an important element of future biomarkers for both prognostic and therapy monitoring in melanoma.

## 6. Systemic Inflammatory Response as a Prognostic Factor in Melanoma

Host immunity plays a defining role in anti-tumor defense. A pro-inflammatory response can be associated with a better prognosis [58]. However, the close connection between inflammation and tumorigenesis is well known. Inflammation can contribute to tumor development, angiogenesis, suppression of apoptosis, and even metastasis [59].

### 6.1. Peripheral Blood Cell Count Ratios

Peripheral blood cell count ratios are a relatively new type of inflammatory indicators, including neutrophil-lymphocyte (NLR), platelet-lymphocyte (PLR), and lymphocyte-monocyte (LMR) ratios, and represent easy-to-measure biomarkers that are part of routine blood examination [60]. Wagner et al. suggested that a relative neutrophil count >69.7%, relative lymphocyte count ≤17.5% and relative monocytes >6.6%, are associated with reduced overall survival in stage I-III melanoma and could be considered prognostic biomarkers [61].

Data from a multicenter cohort study that included 1489 patients with melanoma, conducted over a 10-year, period showed that there is a positive correlation between NLR and tumor volume upon presentation. In addition, NLR may predict occult sentinel lymph node metastases [62]. NLR > 2 correlates with lymph node metastases and recurrences in patients with acral lentiginous melanoma [59]. Lymphocytes in tumor infiltrates are involved in anti-tumor defense, as aforementioned. CD8+ T cells have the ability to directly destroy tumor cells by releasing perforins and granzymes. CD4+ T cells participate in this process by releasing pro-inflammatory cytokines (TNF alpha, IFN gamma, IL-2). However, the presence of neutrophils in the TME is associated with a poor prognosis. Neutrophils are involved in the process of invasion and metastasis through their ability to induce the degradation of the extracellular matrix. Additionally, neutrophils stimulate the adhesion capacity of malignant cells [63].

Iacono et al. analyzed the LMR in the blood of 162 patients with advanced melanoma and observed that a high LMR is associated with a better prognosis in these patients. Elevated LMR was correlated with normal levels of lactate dehydrogenase (LDH), the absence of metastases in the central nervous system and fewer than three metastatic sites [64]. It should be noted that a high number of circulating monocytes reflects a large amount of macrophages that are attracted to the TME [64]. Jairath et al. conducted a retrospective study that included patients with cutaneous melanoma in the TCGA database and analyzed the immune cell populations in the TME. They showed that a reduced lymphocyte-to-monocyte ratio (LMR) and an elevated amount of undifferentiated macrophages represent negative prognostic factors [65]. Wade et al. raised the hypothesis that decreased NLR and LMR are associated with the regression of primary melanoma [58].

The meta-analysis by Zhang et al., which included nine studies conducted between 2016 and 2019, aimed to evaluate the prognostic value of PLR in melanoma patients and concluded that a high PLR correlates with poor overall survival in melanoma patients [66]. Peripheral blood count was also used to evaluate the effectiveness of immunotherapy in several studies that showed that elevated NLR and PLR are associated with a lower response to immunotherapy [67]. The platelets are activated by the tumor cells that reach the bloodstream and favor the formation of microthrombi that help tumor cells to escape the action of NK cells. In addition, platelets participate in tumor progression and metastasis. Malignant cells present CD97, which induces the release of lysophosphatidic acid from platelets, resulting in transendothelial migration. Additionally, platelets can exert an inhibitory effect on T cell activity by activating TGF beta [68]. In a mouse model, platelets promoted melanoma dissemination to the lungs [68].

The red blood cell distribution width (RDW) is defined as the variation in the volume of red blood cells. Increased RDW is specifically associated with anemia, chronic inflammation and malnutrition, characteristics of advanced stages of cancer [69]. Moreover, increased values of RDW represent a negative prognostic factor in several types of cancer, including esophageal, colorectal, gastric etc. [70,71,72]. In fact, RDW reflects the presence of inflammation produced by the TME and malignant cells. It is known that inflammation inhibits the synthesis of erythropoietin and the release of iron from reticuloendothelial macrophages [72]. Hannarici et al. analyzed 114 patients with melanoma and showed that the RDW to lymphocyte ratio (RLR) is an independent prognostic factor for overall survival and progression-free survival in patients with cutaneous melanoma [73].

### 6.2. Eosinophils

The link between eosinophils and cancer was discussed for the first time approximately 100 years ago, but many elements remain unknown. The accumulation of eosinophils was observed in the peripheral blood of patients with cancer and in the TME. Cancer cells release molecules with a chemoattractant effect on eosinophils, among the most important being IL-5 and GM-CSF [74]. Using a murine model, Tepper et al. showed that eosinophil degranulation had a defining role in the elimination of metastases [75]. Eosinophils exert an antitumor effect; they regulate the angiogenesis process, which is essential for tumor growth, promote the normalization of tumor vessels, modulate inflammatory cell activity in the TME and exert a cytotoxic effect on malignant cells by releasing compounds such as major basic protein (MBP), eosinophil cationic protein (ECP), eosinophil-derived neurotoxin (EDN), TNF-α, and granzymes [74]. However, there are studies that suggest the protumor effect of eosinophils [76,77]. The stimulatory effects of eosinophils on tumor progression could be influenced by certain TME-related factors [74].

Pereira et al. have shown that patients with metastatic melanoma who have an increased number of circulating eosinophils have a better prognosis, regardless of the therapy they receive [78]. Regarding eosinophil-cationic protein (ECP), the data are interesting. ECP is released by activated eosinophils and has several functions, including mast cell degranulation, inhibition of immunoglobulin synthesis, suppression of T cell proliferation, and modulation of fibroblast activity. ECP can lead to tumor infiltration through muscle fiber corrosion [79]. Kruckel et al. proposed the serum level of ECP as a biomarker in metastatic melanoma. Their study showed that the increase in serum ECP levels is correlated with a lower survival rate, the correlation being independent of the presence of eosinophilia or therapy. No correlation was identified between ECP and LDH [80]. Biomarkers such as eosinophil count and serum levels of ECP can be significantly associated with good survival in advanced-stage melanoma patients [81].

## 7. New Insights into Angiogenesis in Melanoma

Angiogenesis is essential for tumor development, progression and metastasis and represents an unfavorable prognostic marker for a variety of tumors. Rapid cell proliferation increases the need for oxygen and nutrients, leading to hypoxia, which is perceived by the immune system as a wound site. In these conditions, immune cells interact with tumor cells and stimulate the release of growth factors, cytokines and chemokines that contribute to the initiation of angiogenesis. Vascular endothelial growth factor (VEGF) is one of the most important factors associated with angiogenesis in melanoma, being involved in endothelial cell proliferation and vascular remodeling [82]. However, recent studies have brought important information to our attention regarding other angiogenesis pathways and proangiogenic factors in melanoma.

IL13Rα2 is a cell surface tumor antigen that has been identified in numerous tumors. IL13Rα2 promotes the activation of several pathways, such as MAPK, Akt/PKB, and Wnt/β-catenin, that induce cell invasion; therefore, the increased expression of IL13Rα2 was associated with an unfavorable prognosis in several neoplasms [83]. Okamoto et al. pointed out that IL13Rα2 is involved in angiogenesis in melanoma, promoting tumor development. They demonstrated that overexpression of IL13Rα2 in patients with melanoma led to increased expression of amphiregulin, a component of the EGF family and a proangiogenic factor, resulting in the stimulation of angiogenesis and tumor growth. However, IL13Rα2 induced a minimal increase in the VEGF level, and moreover, loss of IL13Rα2 expression in the A375 melanoma cells did not change the level of VEGF [84]. Monteiro et al. revealed, on murine melanoma cell lines, that there is a positive correlation between the expression of the angiogenic factors, *Vegfc*, *Angpt2*, and *Six1* and the degree of aggressiveness of melanoma cells. Furthermore, using data from TCGA, they observed that the angiogenic factor *ANGPT2* and the lymphangiogenic receptor *VEGFR-3* can be considered independent factors to predict overall survival in patients with melanoma [85]. In line with this, Abdul Pari et al. observed that an increased number of metastatic melanoma samples expressed ANGPT2 compared to primary melanomas or nevi. The results were completed by analyzing Human Protein Atlas datasets, and a negative correlation was observed between ANGPT2 expression levels and overall survival in melanoma patients [86]. 

Urokinase plasminogen activator receptor in overexpressed in numerous cancers and is associated with an aggressive phenotype of the tumor [87]. The study by Hugdahl et al. showed that urokinase plasminogen activator receptor (uPAR) could represent a marker of vascular proliferation and prognosis in melanoma. A positive correlation was identified between uPAR expression and melanoma thickness, the presence of necrosis/ulceration and mitoses. In addition, increased uPAR expression correlates with a poor outcome in primary melanoma [88]. Angiogenesis process and its traits in melanoma can be an important source of validated biomarkers.

## 8. Proteins, Enzymes and Post-Translational Modifications as Biomarkers 

### 8.1. Aberrant Glycosylation in Melanoma

Glycosylation is a posttranslational modification of proteins that is involved in the regulation of numerous processes, such as protein folding and degradation or the interaction between cells and the extracellular matrix. Altered protein glycosylation leads to the stimulation of oncogenic signaling pathways and loss of intercellular adhesion, favors cell migration and dissemination, and induces a pro-metastatic profile [89]. Aberrant glycosylation represents a hallmark of cancer. In melanoma, the most frequent glycosylation changes are sialylation, fucosylation, and N- and I-glycan branching [89]. Recent studies have drawn attention to the incompletely elucidated role of the glycome in melanoma, which comprises the glycans and glycoconjugates produced by the cells of an organism. Aberrant cell-surface glycosylation is a major characteristic of tumor cells, reflecting cancer-specific changes in glycan biosynthesis pathways; therefore, researchers have investigated the glycosylation process in melanoma, focusing mainly on glycosyltransferases, the key enzymes involved in glycan synthesis [90].

Glycosyltransferases add specific glycans to proteins, while glycosidases are involved in the trimming of glycans, and are found in the endothelial reticulum and Golgi apparatus, where they participate in the synthesis and processing of the glycans to obtain their final structure [90]. According to recent research, I-antigen-forming β1,6 N-acetylglucosaminyltransferase 2 (GCNT2) was proposed as a potential novel biomarker for melanoma progression. In melanoma, GCNT2 inhibits tumor proliferation, in contrast to the effect observed in other cancers, where it is involved in tumor invasion [90,91]. In line with this, the GEO data analysis showed that the glycosyltransferase 8 domain containing 1 (*GLT8D*)1 expression was increased at the mRNA level in melanoma compared to nevi. Using immunohistochemical analysis, it was highlighted that GLT8D1 has tissue specificity. The expression of the GLT8D1 protein is higher in both cutaneous and mucosal melanoma than in nevi. In addition, GLT8D1 protein expression was higher in cutaneous melanoma compared to mucosal melanoma. When acral melanoma samples were compared with non-acral melanomas, the expression was higher in acral melanoma. Important correlations were identified between the high GLT8D1 protein expression and Clark level, AJCC stage, the presence of ulceration, Ki-67 expression and histopathological type [92].

Shan et al. used C8161 cells (high metastatic human melanoma cells) and A375P cells (low metastatic human melanoma cells) to evaluate the differences in glycosylation. They highlighted that the composition profile of fucosylated N-glycans was different in the two types of cells, and the expression of fucosyltransferase (FUT) 4 mRNA was higher in C8161 cells. Furthermore, aberrant expression of FUT 4 was associated with alterations in the migration and invasion capacity of melanoma cells by regulating the PI3K/Akt signaling pathway; therefore, the authors proposed FUT 4 as a regulatory marker of these processes in melanoma and a biomarker for detecting metastatic melanoma [93]. When comparing melanoma patients with healthy controls, Virag et al. observed that there are differences in the glycan composition of human serum alpha-1-acid glycoprotein (AGP) between the two groups. In patients with melanoma, there was an increased level of fucosylated glycans and a low level of their non-fucosylated counterparts. Thus, the authors concluded that the glycosylation pattern of human serum AGP could represent an important biomarker for the diagnosis of melanoma with a better sensitivity than serum S100B [94]. Table 2 summarizes the glycosylation deregulations that can be further validated in melanoma.

### 8.2. TBC Proteins as Biomarkers in Melanoma

The TBC domain is a protein domain found in all eukaryotes and is named according to its initial discovery in the proteins Tre-2, Bub2, and Cdc16 [95]. TBC family members regulate the cell cycle and over 60 Rab GTPases have been described in mammals and represent the largest class of the Ras-related GTPases [96]. Rab proteins are involved in processes such as vesicle fusion, membrane transport and cell division [97]. In humans, approximately 40 TBC domain-containing proteins have been described and represent a group of proteins that modulate the small GTPases and operate through the interactions with Rab-like small G proteins [96,98]. It has been reported that a part of them is involved in carcinogenesis. For example, the expression of the oncogene *TRE17* was observed in Ewing sarcoma [99] and in lung cancer, it was shown that cell proliferation is inhibited by siRNA-mediated knockdown of TBC1D7 [97].

Considering that TBC1D16 has been previously shown to be involved in melanoma metastasis, Qi et al. studied whether other TBC proteins are involved in this process and identified the upregulation of TBC1D4, TBC1D14, TBC1D16, TBC1D7, and TBC1D10A and the downregulation of TBC1D24 proteins. They found an important correlation between TBC1D7 and distant metastasis-free survival. Using cultured melanoma cells, (primary and metastatic melanoma cell lines derived from the same patient), the authors highlighted that TBC1D7 increases cell invasion by influencing the activity of MMP2 and MMP9 [98]. Tang et al. identified a group of seven TBC genes (*TBC1D13*, *TBC1D16*, *TBC1D7*, *TBC1D8B*, *TBC1D15*, *TBC1D19*, and *TBC1D10C*) as prognostic markers in melanoma. They compared the transcription levels of these genes with the TCGA melanoma dataset and observed that the relative mRNA expressions of TBC1D10C, TBC1D19, TBC1D16, TBC1D13 and TBC1D7 were higher and expression of TBC1D15 was lower, while for TBC1D8B, there were no differences between the melanoma samples and normal tissues. Based on these genes, the authors developed a prognostic nomogram model for predicting survival in melanoma. They also showed that the alteration of TBC1D7 hinders the proliferation and dissemination of tumor cells in vitro [100]. TBC proteins can represent another tissue biomarker to be considered in melanoma management.

### 8.3. The Role of Nicotinamide N-Methyltransferase in Melanoma

Nicotinamide N-methyltransferase (NNMT) is an enzyme that catalyzes the N-methylation of nicotinamide, and structurally related compounds; it uses S-adenosylmethionine (SAM) as a methyl donor and is responsible for the modulation of intracellular concentration of nicotinamide. NNMT is a cytosolic enzyme, which, under physiological conditions, is expressed especially in the liver and adipose tissue and in low amounts in the brain, skeletal muscles, kidneys, heart and placenta. Dysregulation of NNMT activity has been associated with numerous conditions, including diabetes mellitus, liver disease, neurodegenerative disorders, and cancer [101,102].

Altered levels of NNMT have been detected in various neoplasms, such as gastric, colorectal, pancreatic, etc. [103]. NNMT is involved in the carcinogenesis process, promoting tumor progression through two main mechanisms, by altering cellular metabolism and epigenetic remodeling. NNMT expression is upregulated or downregulated in different tumors. Increased expression of the enzyme is correlated with cell proliferation and invasion in some types of neoplasia, and low expression in other tumors is considered a mechanism to preserve the specific tumor cell phenotype [104].

Ganzetti et al. evaluated the role of NNMT in the diagnosis and prognosis of melanoma by analyzing the expression of the enzyme through immunohistochemical analysis of 34 melanomas and 34 nevi (control group). NNMT expression in melanoma was statistically significantly higher compared to nevi. An inverse correlation was identified between the enzyme levels and Breslow thickness, Clark level, mitotic count, and the presence of ulceration [103]. In another study, NNMT activity was analyzed by shRNA-mediated silencing of the enzyme in the melanoma cell lines A375 and WM-115 and it was found that downregulation of NNMT induced a decrease in the proliferation and migration of melanoma cell lines. Furthermore, lower enzyme activity leads to a higher susceptibility of cancer cells to dacarbazine, suggesting the possible involvement of NNMT in the mechanisms that underlie the development of chemoresistance [105]. Mascitti et al. showed that NNMT expression was different in cutaneous melanoma compared to oral melanoma. Immunohistochemical analysis indicated a higher extension of NNMT expression in cutaneous melanoma samples, but oral melanoma samples were characterized by a higher expression in tumor cells. Regarding the presence of ulceration, there was a link between ulceration and NNMT expression, but in a different mode in the case of the two types of tumors. The staining intensity was increased in ulcerated samples of oral melanoma, while the samples of cutaneous melanoma, which presented ulceration, exhibited lower NNMT levels [106]. Changes in NNMT expression have also been reported in non-melanoma skin cancers, such as basal cell carcinoma or squamous cell carcinoma [107,108,109].

## 9. Metabolomic Markers in Melanoma

The rapidly emerging field of metabolomics provides novel biomarkers in melanoma [110]. Cancer cell metabolism is modified to allow cell proliferation. The Warburg effect, a hallmark of cancer, implies accentuation of the glycolysis process, leading to increased glucose uptake and lactate excretion, regardless of oxygen availability [111]. Lactate elicits an immunosuppressive environment in melanoma. The transport of lactate by SLC16A1 into the TME leads to increased cellular metabolism and promotes the survival of melanoma cells and could represent a potential prognostic biomarker. In addition, Tregs from the tumor infiltrate that play a critical role in suppressing the immune response use lactic acid. SLC16A1, a member of the SLC transporter superfamily, is highly expressed in melanoma. The roles of the genes co-expressed with SLC16A1 involve the modulation of metabolic pathways, protein ubiquitination, and substance localization. Furthermore, SLC16A1 has been associated with immune cell infiltration [112]. It seems that altering the metabolic phenotype is important in the progression of melanoma. In advanced melanoma, there is an increase in aerobic glycolysis; glyceraldehyde-3-phosphate dehydrogenase (GAPDH) and pyruvate kinase M2 (PKM2) are overexpressed in melanoma compared with nevi, and their expression increases from the radial growth stage to metastatic melanoma. Additionally, the expression of mitochondrial oxidative phosphorylation (OXPHOS) proteins (HSP60) is increased and β-F1-ATPase is downregulated. However, at the same time, the bioenergetic cellular (BEC) index is low. The BEC index is calculated according to the following formula: β-F1-ATPase/HSP60/GAPDH ratio and is a marker of glucose consumption by tumor cells. Thus, the dysregulation of mitochondrial and glycolytic proteins and the decrease in BEC index are characteristics of aggressive tumors and are associated with tumor dissemination [113].

Numerous metabolic pathways are altered in melanoma [111,114]. Kim et al. showed through metabolomic analysis that aminomalonic acid, which can be generated by free radical damage to proteins, could represent a biomarker to differentiate low metastatic melanoma from high metastatic melanoma. Lipid analysis indicated that phosphatidylinositol species with saturated and monounsaturated fatty acyl chains could also be used to assess the metastatic capacity of melanoma [115]. Changes in lipid metabolism represent an important feature of aberrant cell proliferation and contribute to tumor progression. Cancer cells have the ability to activate adipocytes that, through lipases, cleave triglyceride deposits, providing fatty acids for cancer cells. Moreover, CAFs can participate in the secretion of lipids and lipid signaling and induce the activation of mitogenic and migratory pathways. Accumulated fatty acids can affect immune cell functions in the TME, exerting a tumor promoting effect, which is responsible for cell resistance to therapy and metastasis [116].

Wasinger et al. showed that glutamic acid and alanine stimulate the proliferation and migration of melanoma cells in the early stages of evolution. This effect was not observed in the case of metastatic cells [117]. Altered levels of alanine, aspartate, and glutamate seem to be involved in the development of melanoma [115,118]. Bayci et al. recently proposed five serum metabolites, one phosphatidylcholine (PC aa C40:3), three acylcarnitines (DL-carnitine, octanoyl-L-carnitine and methylmalonyl-L-carnitine) and ethanol as biomarkers for advanced melanoma [119]. Kosmopoulou et al. analyzed the metabolic profile of the cell lines WM115 and WM2664, derived from the same patient. WM115 was derived from a primary tumor and WM2664 was derived from lymph-node metastases. The study results revealed the important role of the metabolism of purines, pyrimidines and amino acids in the metastasis process. WM115 showed elevated levels of phosphocholine, choline, guanosine and inosine. On the other hand, WM2664 exhibited elevated levels of hypoxanthine, myo-inositol, glutamic acid, organic acids, purines, pyrimidines, AMP, ADP, ATP and UDP(s) [120]. Purine synthesis is an absolutely necessary event for cell proliferation and metastasis [111]. Metabolomics offers new research avenues in melanoma and by analyzing tumor cell metabolism and immune cell metabolism, new biomarkers can emerge.

## 10. Conclusions

Melanoma is a multifaceted tumor with a great ability of invasion and metastasis, characterized by high genomic, proteomic, epitranscriptomic, and metabolomic versatility. A significant number of biomarkers from many sources, including the TME, tumor metabolism, aberrant glycosylation, miRNAs and host immune response, have been analyzed and show promising results for the diagnosis and prognosis of melanoma. The investigation of a complex panel of biomarkers in melanoma is currently in progress, and further studies are required to establish the most reliable biomarkers.

## Figures and Tables

**Table 1:** Summary of the novel miRNAs or miRNA panels in melanoma.

miRNA/miRNA Panels	Function/Significance
miR-431	Downregulates cell proliferation, migration and invasion via NOTCH2 [33]
miR-107	Inhibits cell growth, migration and invasion via POU3F2 [34]
miR-135b	Promotes cell proliferation and migration via LATS2 [35]
miR-424	Associated with decreased overall survival [36]
miR-25, miR-204, miR-211, miR-510, miR-513c	Regulate genes related to PI3K-Akt pathways, ubiquitin-mediated proteolysis and focal adhesion [37]
miR-142-5p, miR-550a, miR-1826, and miR-1201	Involved in the development of primary melanoma [39]
miR-182-5p, miR-199a-5p, miR-877-3p, miR-1228-3p and miR-3613-5p	Associated with micrometastatic regional lymph node disease [40]
exo-miRNA-532-5p and exo-miR-106b	May be used to identify patients with early-stage melanoma [42]
exo-miR-1180-3p	Negatively corelates with melanoma cell proliferation [41]

NOTCH2—neurogenic locus notch homolog protein 2, POU3F2—POU class 3 homeobox 2, LATS2—large tumor suppressor kinase 2.

**Table 2:** Novel markers of aberrant glycosylation in melanoma.

Glycosylation Deregulation	Significance
Loss of GCNT2	Promotes melanoma growth and stimulates cell survival [91]
Overexpression of GLT8D1	Correlates with Clark level, AJCC stage, the presence of ulceration, Ki-67 expression and histopathological type [92]
Overexpression of FUT 4	Enhances the migration and invasion of melanoma cells [93]
Altered glycosylation pattern of human serum AGP	An increased level of fucosylated glycans and a low level of their non-fucosylated counterparts in melanoma patients compared to healthy subjects [94]

GCNT2—β1,6 N-acetylglucosaminyltransferase 2, GLT8D1—glycosyltransferase 8 domain containing 1, FUT 4—fucosyltransferase 4; AGP—alpha-1-acid glycoprotein.

## Data Availability

Not applicable.
